# Proceedings of the Quarterly Meeting of the De Witt County Medical Society, Held at Mt. Pleasant, Ill. Jan. 5th, 1857

**Published:** 1857-02

**Authors:** 


### MEDICAL INTELLIGENCE.

*Proceedings of the Quarterly Meeting of the De Witt County Medical Society, held at Mt. Pleasant, Ill. Jan.* 5, 1857.

The Society met in Odd Fellows' Hall, Mt. Pleasant, Ill.

The President, Dr. C. Goodbrake, of Clinton, called the Society to order at 2 o'clock P.M.

The Secretary (Dr. Madden) being absent, Dr. E. Richards, of Mount Pleasant, was appointed Secretary *pro tem.*

The minutes of the last meeting were read and approved.

On motion, Drs. Stephen W. Noble, of Le Roy, Ill. John McHugh, of Waterloo, Iowa, and Dr. Andrews, of New York, who were present, were invited to participate in the proceedings of the meeting.

The reading of essays being in order, Dr. Richards read a very interesting and well-written paper on Mercury. The essayist first described its properties as a metal; then some of the different preparations of it, also their value as therapeutic agents—dwelling more particularly upon blue mass and calomel, as being the two preparations most frequently employed; and contended that neither of these preparations, as such, produced the specific effects of mercury on the system, but that they were converted in the digestive organs into corrosive sublimate previous to their being absorbed.

The essay called forth a spirited debate in which Drs. Noble, McHugh, Brown, Adams, Lewis, Richards, and Goodbrake participated. During the debate Dr. McHugh asked the question, Is the administration of calomel indicated in typhoid fever?

Dr. Lewis always endeavored to produce *slight* ptyalism in the commencement of the disease; and when he could produce this effect, he almost invariably succeeded in cutting short the disease. But he had in some instances given it every three hours, for two or three weeks, without being able to affect the gums.

Dr. Goodbrake deprecated the general use of calomel in the disease under consideration. He sometimes prescribed it in the commencement of the disease, more rarely in its advanced stages, when symptoms supervened indicating its use: but never, intentionally, to the extent of ptyalism, and never with the view of treating typhoid fever, *as a disease*, with the medicine in question.

Dr. Adams read a very able and ingeniously written paper on the *modus operandi* of Quinine; which called forth another very interesting discussion, in which most of the gentlemen present partook. Pending the discussion, the Society adjourned until 7 o'clock P.M.

EVENING SESSION.

The President called the Society to order at 7 o'clock P.M.

The debate upon the peculiar views of the essayist, in relation to the modus operandi of quinia, was continued until a late hour.

The President appointed Dr. Edmiston, of Clinton, and Dr. Brown, of Mt. Pleasant, to write each an essay on some medical subject of their own choice, to be read at the next meeting of the Society.

On motion of Dr. Brown, it was

*Resolved*, That the thanks of the Society be tendered to Drs. Richards and Adams for their essays.

On motion of Dr. Adams, the following resolution was adopted:—

*Resolved*, That the thanks of this Society are due and are hereby tendered to the Odd Fellows of Mt. Pleasant for the use of their Hall.

Dr. Adams offered the following resolution, which was unanimously adopted:—

*Resolved*, That the thanks of this Society be, and are hereby returned to Drs. Richards and Brown, for the sumptuous dinner they had served up for the Society, at the Gardener House.

On motion the Society adjourned to meet in annual session at Clinton, on the first Monday in April, at 10 o'clock A.M.

C. GOODBRAKE, M.D. *President.*

E. RICHARDS, *Secretary, Pro Tem.*

